# Reduced Expression of the *SHORT-ROOT* Gene Increases the Rates of Growth and Development in Hybrid Poplar and Arabidopsis

**DOI:** 10.1371/journal.pone.0028878

**Published:** 2011-12-14

**Authors:** Jiehua Wang, Sara Andersson-Gunnerås, Ioana Gaboreanu, Magnus Hertzberg, Matthew R. Tucker, Bo Zheng, Joanna Leśniewska, Ewa J. Mellerowicz, Thomas Laux, Göran Sandberg, Brian Jones

**Affiliations:** 1 College of Agriculture and Bioengineering, Tianjin University, Tianjin, China; 2 Umeå Plant Science Centre, Department of Plant Physiology, Umeå University, Umeå, Sweden; 3 Umeå Plant Science Centre, Department of Forest Genetics and Plant Physiology, Swedish University of Agricultural Sciences, Umeå, Sweden; 4 Faculty of Biology III, University of Freiburg, Freiburg, Germany; 5 Faculty of Agriculture, Food and Natural Resources, University of Sydney, Sydney, Australia; Kyushu Institute of Technology, Japan

## Abstract

SHORT-ROOT (SHR) is a well characterized regulator of cell division and cell fate determination in the *Arabidopsis* primary root. However, much less is known about the functions of SHR in the aerial parts of the plant. In this work, we cloned *SHR* gene from *Populus trichocarpa* (*PtSHR1*) as an *AtSHR* ortholog and down-regulated its expression in hybrid poplar (*Populus tremula*×*P. tremuloides* Michx-clone T89) in order to determine its physiological functions in shoot development. Sharing a 90% similarity to AtSHR at amino acid level, PtSHR1 was able to complement the *Arabidopsis shr* mutant. Down regulation of *PtSHR1* led to a strong enhancement of primary (height) and secondary (girth) growth rates in the transgenic poplars. A similar approach in *Arabidopsis* showed a comparable accelerated growth and development phenotype. Our results suggest that the response to SHR could be dose-dependent and that a partial down-regulation of *SHR* could lead to enhanced meristem activity and a coordinated acceleration of plant growth in woody species. Therefore, SHR functions in plant growth and development as a regulator of cell division and meristem activity not only in the roots but also in the shoots. Reducing SHR expression in transgenic poplar was shown to lead to significant increases in primary and secondary growth rates. Given the current interest in bioenergy crops, SHR has a broader role as a key regulator of whole plant growth and development and SHR suppression has considerable potential for accelerating biomass accumulation in a variety of species.

## Introduction

Significant improvements in forest and energy crop productivity are essential if we are to meet the growing demands for bioenergy and biomaterials [1], [2]. Plant growth is derived predominantly from cells produced in indeterminate meristems [3]. In herbaceous species like *Arabidopsis thaliana*, the majority of cells are produced in meristems of the root and shoot apices. In trees and other woody species, the vascular cambium (VC), a specialized, cylindrical meristem, provides the cells for increases in girth. Although these meristems are structurally and functionally distinct, common elements contribute to their regulation [4]. For example, in *Arabidopsis*, two related homeobox genes, *WOX5* (*WUSCHELRELATED HOMEOBOX 5*) and *WUS* (*WUSCHEL*) are critical for root apical meristem (RAM) and shoot apical meristem (SAM) maintenance, respectively [5].

SHORT-ROOT (SHR) is a putative transcription factor of the GRAS family and is a key component in a developmental pathway regulating the specification of the root stem cell niche as well as the radial patterning of the root in *Arabidopsis* [6], [7]. In trees, the *Pinus radiata SHORT-ROOT* (*PrSHR*) gene has also been shown to play a role in root meristem formation and maintenance and in the cambial region of hypocotyl cuttings [8]. In *Populus trichocarpa*, *PtSHR1* is a close homolog of the *Arabidopsis* RAM regulator, *AtSHR*, and its expression has been detected in the VC of rapidly growing stems of hybrid poplar [9]. Another related protein, SCARECROW (SCR), has a similar role alongside SHR [6] and both of them encode closely related transcription factors belonging to the GRAS gene family [7], [10]. In *Arabidopsis* roots, AtSHR acts, at least in part, non-cell-autonomously, directly and indirectly regulating the expression of a wide range of downstream genes including SCR [10] and a number of cell cycle components [11]. The complete absence of the protein in loss-of-function, *shr* mutant leads to the terminal differentiation of the root apical stem cell niche which organizes centre cells (quiescent centre, QC) and the surrounding stem cells, resulting in the collapse of the RAM and a cessation of longitudinal root growth [6], [12]. AtSHR has also been shown to be essential for the asymmetric periclinal division of a subset of RAM stem cell daughter cells (cortex/endodermal initial, CEI) [10], the specification of the endodermal cell layer in both roots and shoots [6], [10], [13], [14], the cell division and specification during root vascular development [13] and the periclinal divisions of cortex cells during root maturation [15]. Recently, it has been reported that in contrast to their specific roles in cortex/endodermis differentiation and stem cell maintenance in the root, SHR and SCR primarily function as general regulators of cell proliferation in leaves [16]. The spatiotemporal activation of specific cell-cycle genes by SHR/SCR has been shown to be required for proper root pattern formation, providing further evidence of a direct molecular link between these key developmental regulators and genes involved in cell-cycle progression [17]. AtSHR has therefore been shown to be important roles in regulating both cell division and cell fate determination processes.

Contrary to the intensive research of AtSHR in *Arabidopsis* root tissue, much less is known of its specific roles in the shoots. The SAM of *Arabidopsis shr* mutants appears to survive intact, but the shoots are considerably dwarfed compared to wild-type plants [6]. This phenotype and the VC expression of *PtSHR1* suggest that SHR may also play important intrinsic roles in the shoot part of plant. In this study, the down-regulation of *PtSHR1* in hybrid poplar led to an enhancement of meristem activity and an acceleration of vegetative growth and development. Our data suggested that SHR is a common regulator of meristem activity in different plant tissues and organs.

## Materials and Methods

### Plant Growth and Transformation

Hybrid poplar (*Populus tremula* L.×*P. tremuloides* Michx. clone T89) was maintained, transformed and regenerated as previously described [18]. For RNAi plasmid construction, the inverted repeat DNA construct for *PtSHR1* was ligated into the pK7GWIWG2(I) [19] binary vector, transferred to *Agrobacterium tumefaciens* and used to transform hybrid poplar stem segments [18]. Primer sequences for cloning the *PtSHR1* inverted repeat construct were 5′-AACCACCACCATCATCACTATC-3′ and 5′- GCTTTCACCTTCAAATGCTTCC-3′. Primer sequences for the RT-PCR analysis of remnant transcript levels in the *PtSHR1* RNAi lines were: 5′-CATCACCTGACCTTCACTCC-3′ and 5′- GTTCGGATTGTTGTTGGAGAC-3′. For the *PtSHR1pro*:*GFP* construct, the primer sequences used to amplify the *PtSHR1* promoter fragment were 5′-GGAGCAAATCATTACACTGTCATG-3′ and 5′- CGGATGGAGTTTGTTGGGGATGGC-3′. After initiation and establishment of rooting in tissue culture, transgenic poplar lines were grown together with their WT control trees, in a mixture of peat and perlite (5∶1) in a glasshouse under natural light supplemented with metal halogen lamps (18 h light/6 h dark) at 22°C/15°C (day/night). They were watered daily and fertilized once a week with a SuperbaS nutrient solution (Supra Hydro AB, Landskrona, Sweden). The plants were grown for 7–9 weeks during which time their positions within the glasshouse were altered every 2–3 days and their heights and diameters were measured. Typically 8–10 trees of WT trees and transgenic trees were grown in parallel in the greenhouse under the same growing conditions. Arabidopsis plants (Col0) were transformed with the respective constructs by *A. tumifaciens*-mediated floral dip transformation [20].

### Microscopy (Light, Confocal Laser Scanning and Scanning Electron)

For microscopic observation, plant tissues were prepared as butyl-methylmethacrylate resin–embedded, semi-thin sectioned material. For immunolocalizations, the resin-embedded material was prefixed with 100 µM m-Maleimidobenzoyl N-hydroxysuccinimide ester (Sigma) in 25 mM Pipes buffer (Sigma), pH 6.9. It then was fixed in 3.7% formaldehyde, 0.2% (v/v) glutaraldehyde in 25 mM Pipes buffer, pH 6.9, embedded in butyl-methylmethacrylate resin mixture, polymerized with UV light, and sectioned (7 µm). After acetone removal of the resin, the sections were incubated in blocking solution (5% skim milk powder in PBS, 137 mM NaCl, 2.7 mM KCl, 2 mM Na_2_HPO_4_, 2 mM KH_2_PO_4_, pH 7.2 to 7.4) and 1% Tween 20 for 45 min, followed by application of the primary antibody (2 h room temperature or overnight at 4°C) and then washed in 0.1% Tween 20 in PBS. The secondary antibody (conjugated to fluorescein isothiocyanate, FITC) was then applied for 1 h at room temperature. The tissue was then washed extensively and stained with 0.01% toluidine blue O for 1 min to minimize tissue autofluorescence. The sections were mounted in Vectashield (Vector Laboratories) and examined by confocal laser scanning microscopy (CLSM). CLSM was carried out with a Leica TCS SP2 AOBS scanning system mounted on a Leica DM IRE2 inverted microscope employing Leica TCS software (using a water corrected 633 objective, Leica). Excitation wavelengths were set at 488 nm (argon laser) for GFP and FITC and 340–380 nm for the counterstain, calcofluor white. Emission was detected between 505 and 520 nm for GFP, 520 and 550 nm for FITC and 420 and 440 nm for calcofluor white. These signals were detected in separate channels. Images were overlaid with Adobe Photoshop 7.0. Monoclonal antibodies raised against recombinant protein PtSHR1 in rabbit were used to localize PtSHR1. Secondary antibodies were anti-rabbit FITC conjugates (Jackson ImmunoResearch Laboratories) (1∶100 dilution). Control and experimental preparations were processed in parallel and viewed at the same confocal laser scanning microscopy settings. To test for tissue autofluorescence, material was processed without the secondary antibodies. Primary antibodies were also saturated with the antigen at 10 mM as a control. These controls gave minimal background signals that were considered nonspecific.

For the scanning electron microscopy (SEM), whole seedlings were fixed in FAA (3.7% formaldehyde, 50% ethanol, 5% acetic acid), substituted with ethanol, then dried from liquid carbon dioxide by the critical point method [21] using a Polaron E-3000 Critical Point Drying Apparatus (Polaron Equipment Ltd, Watford, UK). All specimens were mounted on conductive carbon tape (Agar Scientific Ltd) and oriented for optimal visualization of the areas of interest and secured with Silver Dag (Agar Scientific Ltd). Surface coating with gold was carried out in a modified vacuum coating system equipped with an automatic tilting and rotating device (Edwards E14 Vacuum Coating Unit, Edwards High Vacuum Ltd) giving a maximal thickness of approximately 15 nm. The prepared specimen were all examined by a Cambridge Instruments Stereoscan 360 iXP Scanning Electron Microscope (Cambridge Scientific Instruments Ltd) operated with standard settings, and micrographs were saved digitally with standardized magnifications.

### GUS Analysis

For detecting β-glucuronidase (GUS) activity, fresh tissue sections or whole tissues were incubated for 4–16 hrs at 37°C with gentle shaking in a substrate solution (1 mM 5-bromo-4-chloro-3-indoyl-beta-D-glucuronide, cyclohexylammonium salt (BioVectra, PEI); 50 mM sodium phosphate buffer, pH 7.2, 0.1% Triton X-100, 1 mM K_3_[Fe(CN)_6_] and 1 mM K_4_[Fe(CN)_6_]). The sections or whole tissues were then fixed for 10 min in a fixing solution (5% formaldehyde, 5% acetic acid and 50% ethanol), washed for several minutes in 50% and 100% ethanol and cleared by an incubation in 0.24 M HCl in 20% methanol at 57°C for 15 minutes followed by 15 min in 7% NaOH in 60% EtOH at room temperature. The sections or whole tissues were then rehydrated in a graded ethanol series (40, 20 and 10% EtOH) and mounted in 50% glycerol. Sections and whole tissues were then examined on an Axioplan 2 microscope and recorded with an AxioVision camera (Zeiss).

## Results

### Down-regulation of PtSHR1 in hybrid poplar leads to an accelerated growth phenotype

**Figure 1:**
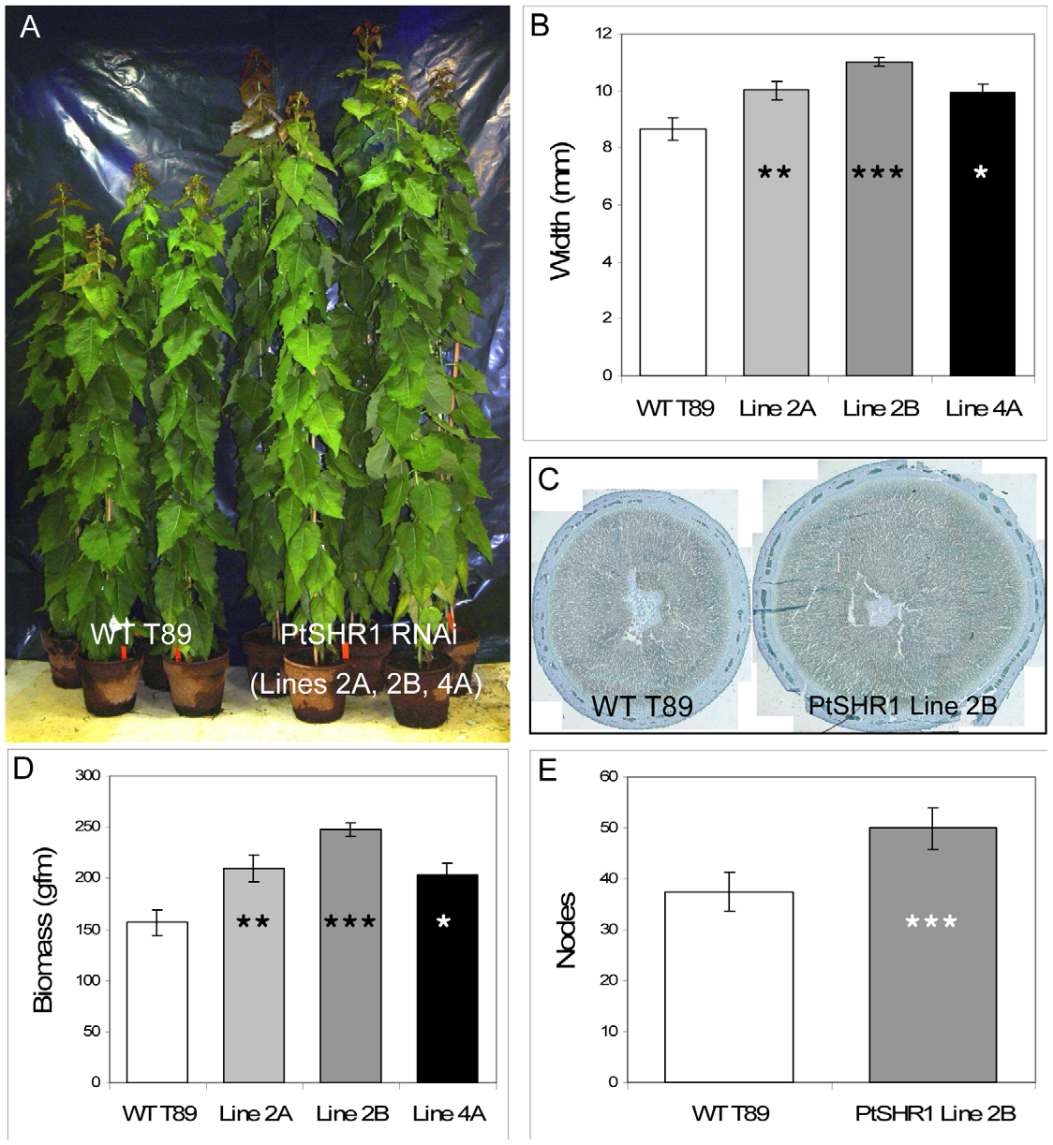
Increased growth of poplar *PtSHR1* RNAi lines after 52 days in the glasshouse. Photo of wild-type hybrid poplar and independent *PtSHR1* RNAi transgenic lines; (A) Average width of stems of *PtSHR1* RNAi lines compared to control lines. Results are average width from 10 internodes beginning at 20 cm above the soil; (B) Transverse sections of wild-type and *PtSHR1* RNAi Line 2B as shown in (B); (C) Average above ground total fresh mass from 20 cm above the soil; (D) Average number of leaves (nodes) in poplar *PtSHR1* RNAi Line 2B stems from 20 cm above the soil to the youngest leaf of at least 4 cm in length; ANOVAs analysis by Dunnett's 2-sided posthoc tests (*n* = 9). The results for *E* were obtained through a *t*-test (*n* = 8). * *P*<0.05, ** *P*<0.01, *** *P*<0.001.

There are three *SHR*-like genes (*PtSHR1*, *PtSHR2A* and *PtSHR2B*) in the genome of *Populus trichocarpa*, however, only *PtSHR1* has the N-terminal domain present in the *Arabidopsis* sequence (Fig. S1). In order to investigate the function of PtSHR1, we generated transgenic hybrid poplar lines (*Populus tremula*×*P. tremuloides*) down-regulated for *PtSHR1* (*PtSHR1* RNAi lines). Schrader *et al.*, (2004) had shown that *PtSHR1* is expressed throughout the cambial zone in poplar tree stems. Given its close homology to *AtSHR*, we hypothesized that reducing the transcript levels of *PtSHR1* would be detrimental to the VC function. Surprisingly, partial suppression of steady-state *PtSHR1* transcript levels by 20–80% (Fig. S2A) resulted in a dramatic acceleration of both primary (Fig. 1A; Table 1) and secondary (Fig. 1B and 1C) growth compared to wild-type (WT) poplar trees. RT-PCR analysis showed that the levels of *PtSHR2A* and *PtSHR2B* transcripts were unaffected in the *PtSHR1* RNAi lines (data not shown). In the strongest RNAi line (Line 2B), *PtSHR1* RNAi trees had accumulated almost 60% more above ground fresh biomass than the equivalent WT trees after approximately two months growth (Fig. 1D). Growth was measured in trees that began as uniform three leaf tissue culture-grown cuttings. Sixteen days after transplantation to soil (DAT), the *PtSHR1* RNAi trees were significantly taller than the WT controls (Table 1). Rather than an increase in internode length, this height advantage in the transgenic trees was the result of an enhanced rate of node production (Fig. 1E).

**Table 1:** ANOVA analysis showing mean height (mm, SDs in parentheses) of independent *PtSHR1* RNAi (Lines 2A, 2B and 4A) and WT T89 lines (^*^ *P*<0.05; ^**^ *P*<0.01; ^***^ *P*<0.001).

	Lines			
Time (Days)	2A	2B	4A	WT
16	216 (17)^**^	239 (25)^***^	205 (33)^*^	176 (23)
28	429 (42)^***^	508 (50)^***^	412 (35)^**^	336 (42)
40	933 (84)^***^	1053 (64)^***^	913 (80)^***^	778 (65)
52	1364 (95)^***^	1497 (57)^***^	1316 (105)^**^	1174 (75)

The main stem tissue and hence the important agronomic resource in poplar and other woody angiosperms is secondary xylem. Its principal components are the xylem fibers. Measured from the apex down in order to compare tissues at the same morphological stage, in young poplar stems there were no significant differences in the amounts of secondary xylem between the *PtSHR1* RNAi and WT trees (data not shown). However, in this young tissue the VC was significantly larger in *PtSHR1* RNAi lines compared to the WT (Table S1). In older stems, the xylem fibers were the main contributor to the differences in girth between the *PtSHR1* RNAi and WT trees (Fig. 1B). As there were no significant differences in the widths or lengths of the individual fibers between the *PtSHR1* RNAi and WT trees (Fig. S3), this increase resulted from enhanced cell proliferation in the cambial zone of the transgenic trees. There were no significant differences in average leaf area or fresh weight between the WT and *PtSHR1* RNAi trees (Fig. S4), indicating that *PtSHR1* suppression leads to a general increases in growth rate and development rather than to specific increases in cell or organ size.

### Shoot growth is affected in *Arabidopsis* loss-of-function *shr* mutants

**Figure 2:**
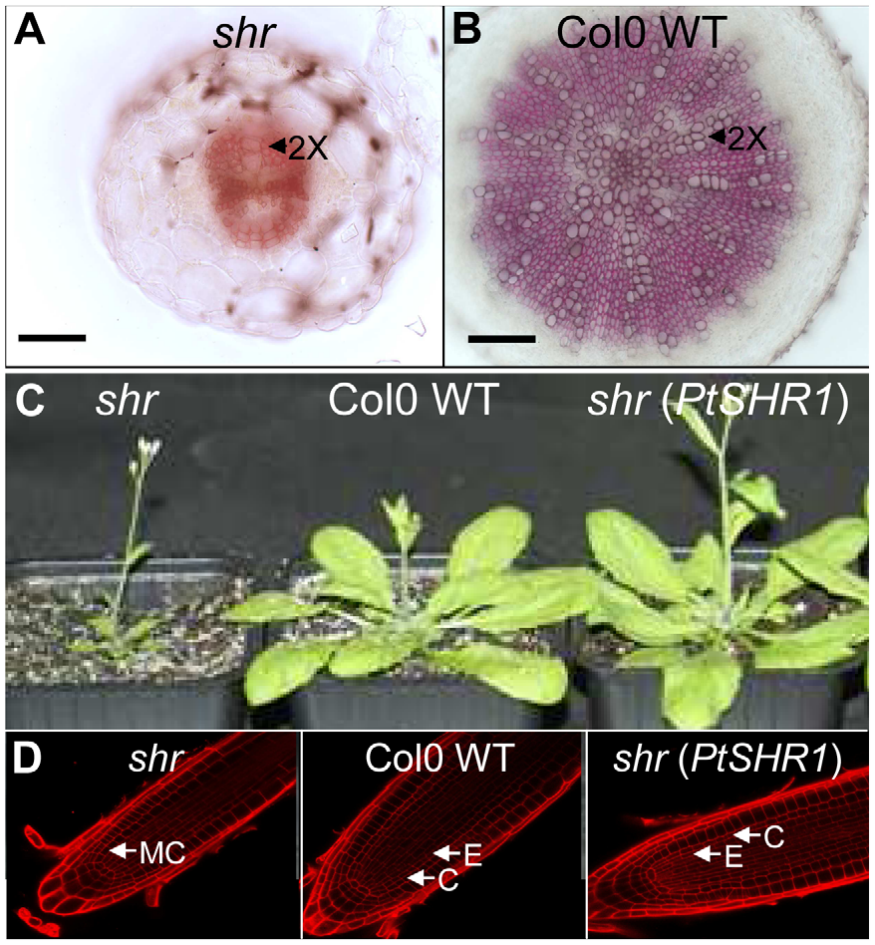
Morphology of *shr*, Col0 wild-type (WT) and prom*AtSHR*:*PtSHR1* complemented *shr* null mutant. Transverse sections of *Arabidopsis shr* mutant hypocotyls at 32 DAS; (A) Col0 WT Arabidopsis hypocotyls at 32 DAS; (B) Shoot phenotype of homozygous *shr* mutant, Col0 WT and homozygous *shr* mutant expressing the *PtSHR1* coding sequence driven by 2.5 kb of the *AtSHR* promoter sequence; (C) Root tips of lines in (C); Hypocotyl sections stained with phloroglucinol to reveal lignified cells. Scale bar is 100 µm. Root tip cell walls stained with propidium iodide. 2×, secondary xylem; MC, mutant cell layer (*shr* lacks normal endodermal cell layer); C, cortex; E, endodermis.

By contrast with the phenotype in the poplar *PtSHR1* RNAi trees, a complete absence of AtSHR in null mutant (*shr*) *Arabidopsis* plants leads to a considerable reduction in the size of the shoot. Fully expanded rosette leaves of *shr* plants are at least 4-fold smaller than the equivalent WT leaves (Fig. S5B) and yet there is no reduction in epidermal cell size (Fig. S5A), indicating a marked reduction in cell division. Despite being an herbaceous annual, a VC is formed in the *Arabidopsis* hypocotyl-root axis [22]. VC activity was considerably reduced in the *shr* mutant compared to the WT (Fig. 2A and 2B), suggesting that, by contrast with PtSHR1 in poplar, AtSHR promotes meristem activity and vegetative growth in *Arabidopsis* shoots. We considered that these opposite effects might be explained in two ways, either by divergent function of AtSHR and PtSHR1 or by a fundamental difference between the complete absence and partial reduction of *SHR* levels.

### *PtSHR1* functionally complements *AtSHR* and have similar expression patterns as *AtSHR*

**Figure 3:**
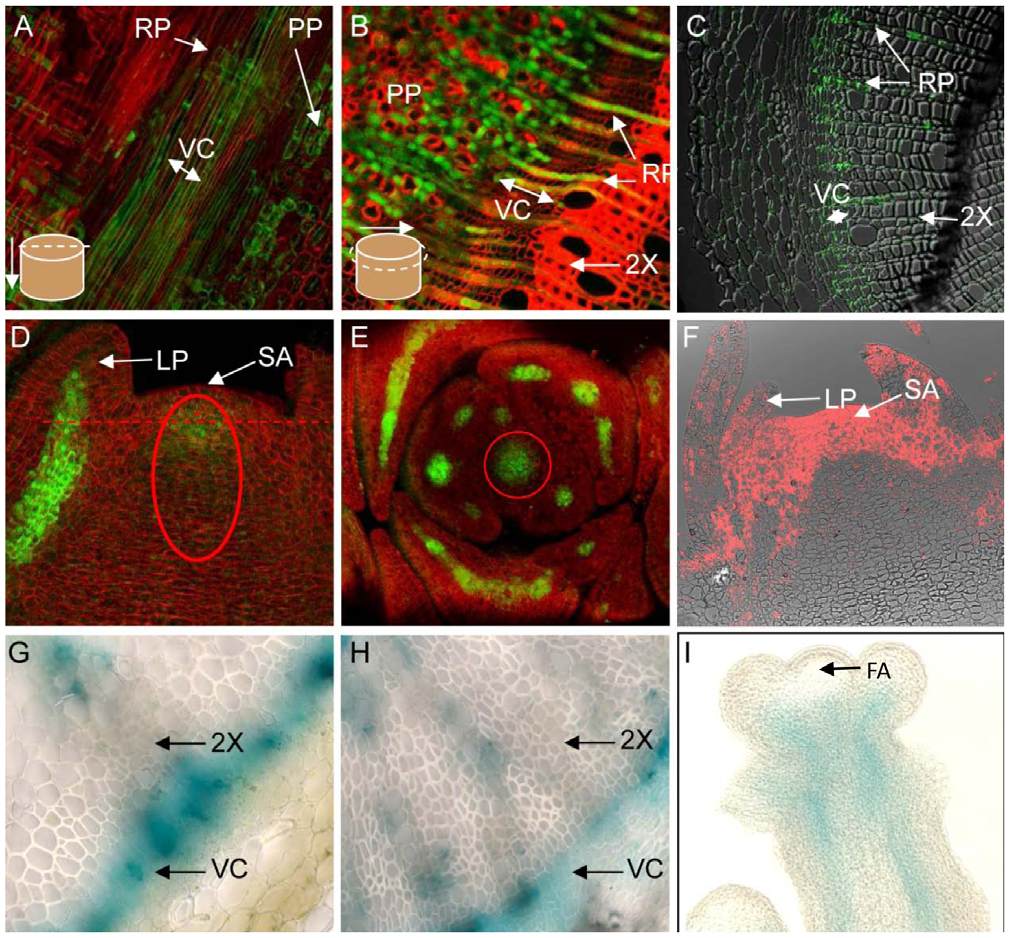
SHR transcriptional activity revealed by prom*PtSHR1::GFP* and *promAtSHR::GUS* expression pattern in poplar (T89) and *Arabidopsis* (Col0), respectively. (A) Tangential longitudinal section through fully elongated, rapidly growing poplar stem; (B) Transverse section through rapidly growing poplar stem; (C) Transverse section through poplar stem showing immunofluorescent labeling (green signal) indicating PtSHR1 epitopes; (D) Radial longitudinal section through poplar apex; (E) Transverse section through apex (taken at position of dotted line in D); (F) Radial longitudinal section through apex showing immunofluorescent labeling (red signal) indicating PtSHR1 epitopes; (G) Transverse section through base of *Arabidopsis* floral stem; (H) Transverse sections though hypocotyl of flowering *Arabidopsis* plant; (I) Radial longitudinal section through *Arabidopsis* floral stem. Oval in (D) showing equivalent cells in circled section in (E). Cell walls in (A), (B), (C) and (D) were counterstained with calcofluor white. RP, ray parenchyma; PP, phloem parenchyma; VC, vascular cambium; 2× secondary xylem; LP, leaf primordium; SA, shoot apex; FA, floral apex.

To consider these two possibilities, we first analyzed whether the PtSHR1 protein could act in the context of the *shr* mutant. Expression of the *PtSHR1* coding sequence, driven by 2.5 kb of the *AtSHR* promoter, rescued the *Arabidopsis shr* mutant shoot (Fig. 2C) and root (Fig. 2D) phenotypes [6], [10], providing strong evidence that PtSHR1 and AtSHR have similar functions. We also observed that both *PtSHR1* and *AtSHR* have remarkably similar expression patterns in their respective species. In poplar roots, a 2.5 kb *PtSHR1* promoter sequence drove GFP expression in the stele in the root tip and in developing lateral roots (Fig. S6), consistent with the published *Arabidopsis AtSHR* root expression data [10]. Transverse and radial longitudinal sections from actively growing two month old poplar stems revealed *PtSHR1pro*-driven GFP throughout the VC (Fig. 3A), in the adjacent phloem axial parenchyma and in the ray parenchyma files leading into the developing secondary xylem (Fig. 3B). Immunofluorescence labeling with a monoclonal anti-PtSHR1 antisera indicated the presence of PtSHR1 epitopes in cells similar to those observed in the *PtSHR1pro*:*GFP* lines (Fig. 3C). Antibody labeling was completely inhibited by a competitor PtSHR1 polypeptide (data not shown). Similarly, in the shoot apex, *PtSHR1pro*-driven GFP was observed in a group of cells in the central zone below the tunica and in provascular tissues of the leaf primordia (Fig. 3D and 3E). Interestingly, immunofluorescence labeling revealed the presence of PtSHR1 epitopes throughout the apical dome and flanking tissues (Fig. 3F). In *Arabidopsis*, a 2.5 kb *AtSHR* promoter sequence drove β-glucuronidase (GUS) expression in the root (Fig. S7) which was consistent with previously published *Arabidopsis AtSHR* root expression data [10]. As we had observed in the poplar *PtSHR1pro*:*GFP* lines, *AtSHRpro*-driven GUS expression was found in the VC of *Arabidopsis* inflorescence stems (Fig. 3G) and hypocotyls (Fig. 3H), in the leaf primordia and in a central zone of vegetative and floral apex (Fig. 3I).

### Down-regulation of *AtSHR* accelerates vegetative growth and development in *Arabidopsis*

**Figure 4:**
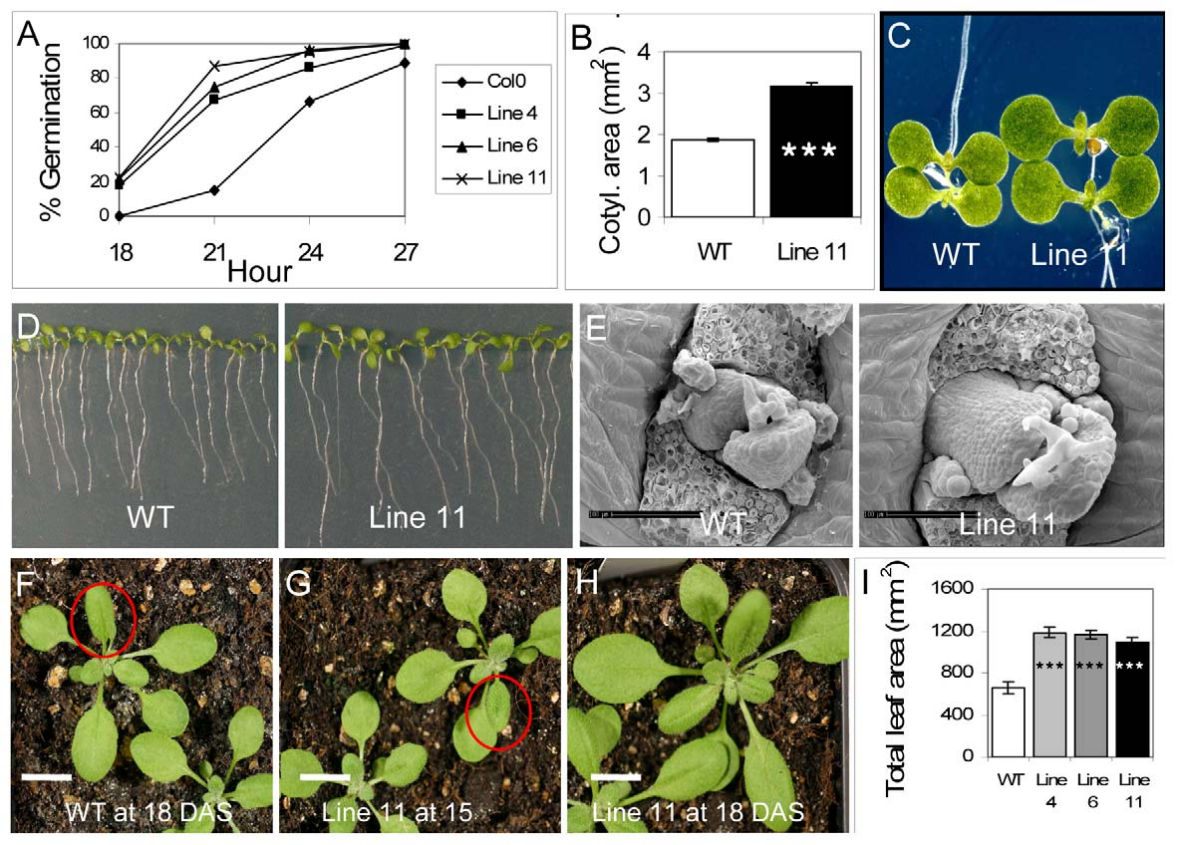
Accelerated growth of *Arabidopsis AtSHR* RNAi lines. (A) Percentage of germination of *AtSHR* RNAi and WT seeds (*n* = 120); (B) *t*-test results comparing WT and *AtSHR* RNAi line 11 cotyledon area at 6 DAS (*n* = 18); (C) *AtSHR* RNAi line 11 cotyledons at 6 DAS; (D) WT and *AtSHR* RNAi line 11 seedlings on vertical agar plates at 6 DAS; (E) WT and *AtSHR* RNAi line 11 showing primordia for leaf 3 and 4 (leaf 1 and 2 removed); (F) WT plants at 18 DAS; (G) *AtSHR* RNAi line 11 plants at 15 DAS; (H) *AtSHR* RNAi Line 11 plants at 18 DAS; (I) ANOVA followed by Dunnett's posthoc test for total area of all leaves at 14 DAS comparing independent *AtSHR* RNAi Lines (4, 6, 11) and WT Col0 (*n* = 8); Scale bar is 100 µm. Red circles in (F) and (G) highlighting leaf 5. *** *P*<0.001.

Given the capacity of *PtSHR1* to rescue the *shr* mutant and their similar expression profiles, we next tested the hypothesis that a reduction, rather than a complete suppression, of *AtSHR* would induce similar effects in *Arabidopsis* to *PtSHR1* suppression in poplar. To do this we generated *AtSHR* RNAi lines with similar levels of down-regulation to the poplar *PtSHR1* RNAi lines (Fig. S2B). As we had observed in the *PtSHR1* RNAi transgenic poplar lines, the *Arabidopsis AtSHR* RNAi plants showed a considerable acceleration in many aspects of vegetative development. The *AtSHR* RNAi lines began to germinate several hours before the WT and continued to develop post germination at an accelerated rate (Fig. 4A–E). When WT seeds were placed at growth temperatures (23°C) earlier than the *AtSHR* RNAi seeds in order to synchronize germination, at 5 days after sowing (DAS) the *AtSHR* RNAi seedlings were on average approximately 50% larger than the WT (*AtSHR* RNAi, 0.68 mg FM/seedling; WT, 0.44 mg FM/seedling). This accelerated development also consistently continued beyond the seedling establishment stage. According to the model for *Arabidopsis* growth stages [23] the WT seedlings reached growth stage 1.06 (six rosette leaves>than 1 mm in length) at 18 DAS (Fig. 4F). The *AtSHR* RNAi plants reached this stage at least three days earlier (Fig. 4G). At 18 DAS, the *AtSHR* RNAi plants were considerably larger than the WT of the same age due to both the increased leaf number and the advanced development of the actively expanding leaves (Fig. 4H and 4I). Once again, as we had observed in the poplar *PtSHR1* RNAi lines, there was no significant (*p*>0.05) difference in fully expanded leaf size between the WT and the *AtSHR* RNAi lines (data not shown). From these above results we hypothesized that plants heterozygous for the *shr* mutation would have a similar phenotype to the *AtSHR* RNAi lines. Indeed, as we had observed in the *AtSHR* RNAi lines, F1 seedlings from crosses between WT (Col0) (male) and *shr* homozygous (Col0) mutant (female) plants developed at an accelerated rate compared to the control [F1 WT (Col0)×WT (312 Col0)] seedlings (Fig. S8).

### Comparison of WT and *AtSHR* down-regulated root apical and shoot apical meristems

**Figure 5:**
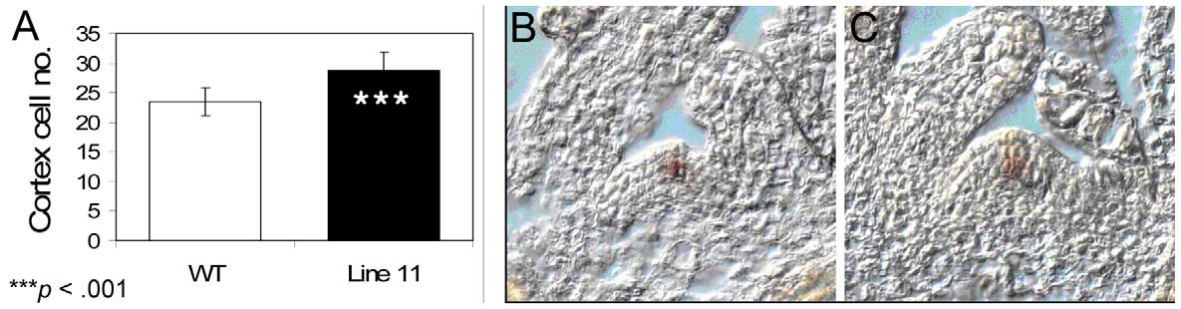
Comparison of WT and RNAi Line 11 root and shoot apical meristems. (A) *t*-test comparing WT with RNAi Line 11 cortex cell number in meristematic zone of root apex 6 DAS. *P*<0.001 (*n* = 33); (B) *in situ* analysis of *CLV3* expression in Arabidopsis vegetative SAMs at 9 days post germination.

As we had seen a larger cambial zone in the stems of *PtSHR1* RNAi poplar trees we examined the RAM and SAM in *Arabidopsis* WT Col0 and *AtSHR* RNAi Line 11 plants to determine whether the down-regulation of *AtSHR* led to similar increases in meristem size. The size of the RAM can be determined by counting the number of root apical cortex cells that have not yet undergone rapid elongation [24]. Consistent with the poplar results, down-regulation of *AtSHR* in *Arabidopsis* led to a significant increase in the size of the RAM (Fig. 5A). SEM images (Fig. 4E and 4F) and longitudinal sections (Fig. 5B and 5C) also indicated that the SAM was larger in the *AtSHR* RNAi plants than in WT plants. Interestingly, *in situ* hybridizations with the SAM stem cell marker, *CLV3* [25], revealed that, relative to the enlarged size of the SAM, the stem cell population remained unchanged (Fig. 5B), suggesting that the down-regulation of *AtSHR* activates cell proliferation concomitantly in the stem cells and their daughters.

## Discussion

AtSHR is known to be essential for the specification and maintenance of the RAM stem cell niche and the radial organization of the *Arabidopsis* root [26], [27]. A complete loss of AtSHR function leads to a differentiation of stem cells, a collapse of the RAM and a cessation of longitudinal root growth [27], [28]. Three SHR-like genes were identified in the poplar genome [9] (Fig. S1). Given the VC expression of *PtSHR1* and its strong homology to AtSHR, we sought to determine the function of the gene in poplar. Expression of the *PtSHR1* coding sequence, driven by the *AtSHR* promoter, rescued the *Arabidopsis shr* mutant phenotype (Fig. 2) providing evidence that PtSHR1 and AtSHR have similar functions. We also observed that both *PtSHR1* and *AtSHR* have remarkably similar expression patterns in their respective species (Fig. 3). However, in contrast to the *Arabidopsis shr* mutant, partial suppression of steady-state *PtSHR1* transcript levels (Fig. S2) resulted in an acceleration of both primary and secondary growth compared to wild-type (WT), T89, trees (Fig. 1 and Table 1). Whereas it is difficult to determine precisely how the downregulation of PtSHR1 led to increased meristem activity, the larger vascular cambium in young stems combined with the meristem expression and protein localization suggest that PtSHR1 is an intrinsic, negative regulator of meristem activity (Fig. 3).

In *Arabidopsis shr* mutants, the RAM terminally differentiates soon after germination leading to an arrest of root growth,whereas the SAM survives intact in the absence of the protein, the shoots of *shr* mutants are considerably dwarfed compared to the WT [6], [12]. Our data showed that this was due to an overall decrease in mitotic activity in the shoot of null *shr* mutants. By contrast, and similarly to the poplar *PtSHR1* RNAi lines, a reduction in *AtSHR* transcript levels in transgenic *AtSHR* RNAi lines and plants heterozygous for SHR led to an increase in meristem activity and an acceleration of growth and development (Fig. 4 and 5). The consistency between the phenotypes of the *AtSHR* down-regulated plants and the poplar RNAi trees reinforces the idea that under normal conditions SHR antagonizes the rates of vegetative growth and development. Combined, the *shr* mutant and *AtSHR* suppression data suggest that the response to AtSHR is dose-dependent but that some level of *AtSHR* expression is required to maintain normal or accelerated rates of meristem activity and growth. Recessive mutations in several genes, including *PLASTOCHRON1* (*PLA1*) [29] and *PLASTOCHRON2* (*PLA2*) [30] in rice, *terminal ear* (*te1*) [31] in maize and *ALTERED MERISTEM PROGRAM1* (*AMP1*) [32] in *Arabidopsis* result in an acceleration of morphogenesis similar to the down-regulation of *SHR*. In homozygous *pla1*, *pla2* mutants the accelerated development is associated with a larger SAM and increased rates of cell division [29], [30]. Similarly, Cockroft *et al.* (2000) showed that a reduction in the length of the cell-cycle G1 phase in transgenic tobacco plants harboring an *Arabidopsis* CycD2 gene under the control of the constitutive cauliflower mosaic virus (CaMV) 35S promoter led to an increased rate of cell division and elevated overall growth rates, increased rates of leaf initiation and accelerated development [33]. Importantly, like the poplar and *Arabidopsis* plants in this study down-regulated for SHR, overall organ size and plant morphology was not affected in the transgenic tobacco plants. From their study, Cockroft *et al.* (2000) suggested that the rate of cell division is the principal determinant of meristem activity and meristem activity in turn determines the overall growth rate of plants [33]. In a work by Dhondt *et al.* (2010), complete elimination of SHR activity in the *shr* mutant led to a reduced rate of cell division and an early exit of cells in developing leaves from the proliferation phase [16]. Transcript profiling experiments have shown that AtSHR regulates root development by modulating the expression of genes involved in a wide range of processes, including signaling, transcriptional regulation and the metabolism and response to the phytohormones, auxin, brassinsteroids (BR) and gibberellic acid (GA) [34]. Recently, it was revealed that *shr* mutants have developmental defects in both protophloem and protoxylem elements, which suggested that SHR plays a central role in the root vascular system to control patterning processes [35]. Although the mechanisms by which SHR regulates the functions of VC and SAM remains to be determined, it is clear from the manuscripts presented here that SHR plays a role determining cell division activity and therefore growth and development of both the root and the shoot.

## Supporting Information

Figure S1 **Comparison of SHR-like sequences.** (A) Multiple sequence alignment of predicted SHR-like amino acid sequences. PtSHR1 (*Populus trichocarpa*), eugene3.01860017, GI: 224114479; *Solanum tuberosum*, TC153082; *Vitis vinifera*, emb_CAN75901.1; *Medicago trunculata*, BG587215; AtSHR, At4g37650; *Oryza sativa*, Os07g0586900; PtSHR2A (*Populus trichocarpa*), eugene3.00070144, GI: 224093015; PtSHR2B (*Populus trichocarpa*), eugene3.00640143, GI: 224133250; *Pinus radiata*, TC60455; *Saccharum officinarum*, TC61564; *Picea glauca*, DV987723; *Glycine max*, TC220926, *Tritcum aestivum*, TC243971; *Zea mays*, TC321921. *Populus trichocarpa* sequences, were obtained from http://genome.jgipsf. org/Poptr1_1/Poptr1_1.home.html; contig sequences, from The Gene Index Project (http://compbio.dfci.harvard.edu/index.html). All other sequences are from GenBank (http://www.ncbi.nlm.nih.gov/). The multiple sequence alignment was performed with Multialin (http://bioinfo.genopole-toulouse.prd.fr/multalin/multalin.html) and ESPript 2.2 (http://espript.ibcp.fr/ESPript/). (B) Comparisons of *Arabidopsis* and poplar SHR-like predicted protein sequences. Phylogenetic analysis of closely related predicted amino acid sequences of *Arabidopsis* and poplar members of the GRAS family [6]. The phylogenetic tree was generated using ClustalW and PHYLIP. The parsimonious tree is shown with bootstrap support values at the nodes. Circles indicate putative poplar-*Arabidopsis* orthology groups. The Gene model IDs from JGI (Joint Genome Institute) or AGI (Arabidopsis Genome Initiative) ID of each sequence are shown in parentheses. *Populus tremula: PtSHR1* (eugene3.01860017), *PtSHR2A* (eugene3.00070144), *PtSHR2B* (eugene3.00640143), *PtSCL35b* (eugene3.00050544), *PtSCL53b* (eugene3.00640007), *PtSCL62* (fgenesh4_pm.C_LG_III000210), *PtSCL69b* (eugene3.00070272), *PtSCL92b* (eugene3.00030248), *PtSCL97b* (eugene3.00011016); *Arabidopsis thaliana*: *AtSHR* (At4g37650), *AtSCL29* (At3g13840), *AtSCL32* (At3g49950).
(DOC)

Figure S2 **Relative transcript levels of WT and independent** ***SHR*** **RNAi suppression lines in poplar and** ***Arabidopsis*** **.** (A) *PtSHR1* RNAi and WT T89 lines. (B) *AtSHR* RNAi and WT Col0 lines. Real-time RT-PCR was used to compare steady-state transcript levels in WT and *PtSHR1* and *AtSHR* in poplar and Arabidopsis, respectively. For poplar, the expression level of 26S proteasome regulatory subunit *S2* was used as an internal reference to which *PtSHR1* expression was normalized. *18S* rRNA was used for Arabidopsi*s*.
(DOC)

Figure S3 **Comparison of fibre widths (A) and lengths (B) comparing independent** ***PtSHR1*** **RNAi (Lines 2A, 2B) and WT T89 lines.** Widths and lengths of fibres extracted from fully elongated internodes of actively growing 2 month old glasshouse grown poplar stems. Means±S.E.M. (*n* = 60). All *P*>0.05 ANOVAs followed by Dunnett's posthoc test.
(DOC)

Figure S4 **Comparison of fully expanded leaf area (A) and fresh weight (B) comparing independent** ***PtSHR1*** **RNAi (Lines 2A, 2B, 4A) and WT T89 lines at 52 days growth in the glasshouse.** Means ±S.E.M. (*n* = 9) are the average area and weight of 15 fully expanded leaves from 20 cm above the soil. All *P*>0.05 ANOVAs followed by Dunnett's posthoc test.
(DOC)

Figure S5 **Comparison of** ***shr*** **mutant with Col0 WT leaf characteristics.** (A) Average leaf epidermal cell area. (B) Total leaf area of fully expanded leaf 4. Means ±S.E.M. Cell area *P*>0.05 (*n* = 20); Leaf area *P*<0.01 (*n* = 10).
(DOC)

Figure S6 ***promPtSHR1*** **(2.5 kb)-driven eGFP expression in poplar (T89) roots.** (A) Expression in stele at root tip. (B) (C) In developing lateral roots. Propidium iodide (red) used as a counterstain.
(DOC)

Figure S7 ***promAtSHR1*** **(2.5 kb)-driven GUS expression in** ***Arabidopsis*** **plants.** (A) Primary root with developing lateral root (circled). (B) Cotyledons and hypocotyl of seedling 16 hours after germination. (C) Fully expanded juvenile rosette leaf. (D) Flower.
(DOC)

Figure S8 **Growth of F1 (WT×WT) and (WT×** ***shr*** **) seedlings.** (A) After 40 hours growth. (B) After 6 DAS. (C) After 15 DAS.
(DOC)

Table S1 *t*-tests showing mean width differences between VC of RNAi lines 2A, 2B, 4A and WT T89. Measurements were taken from images of plastic embedded transverse sections of young stems (18 internodes down from youngest visible leaf primordium) (^*^ *P*<0.05; ^**^ *P*<0.01).
(DOC)
