# Supplementary material for: Reduced Expression of the SHORT-ROOT Gene Increases the Rates of Growth and Development in Hybrid Poplar and Arabidopsis
Source: PLoS One. 2011 Dec 14;6(12):e28878. doi: 10.1371/journal.pone.0028878 (PMC3237562; doi:10.1371/journal.pone.0028878)
Supplement: Figure S1 — Comparison of SHR-like sequences. (A) Multiple sequence alignment of predicted SHR-like amino acid sequences. PtSHR1 (Populus trichocarpa), eugene3.01860017, GI: 224114479; Solanum tuberosum, TC153082; Vitis vinifera, emb_CAN75901.1; Medicago trunculata, BG587215; AtSHR, At4g37650; Oryza sativa, Os07g0586900; PtSHR2A (Populus trichocarpa), eugene3.00070144, GI: 224093015; PtSHR2B (Populus trichocarpa), eugene3.00640143, GI: 224133250; Pinus radiata, TC60455; Saccharum officinarum, TC61564; Picea glauca, DV987723; Glycine max, TC220926, Tritcum aestivum, TC243971; Zea mays, TC321921. Populus trichocarpa sequences, were obtained from http://genome.jgipsf. org/Poptr1_1/Poptr1_1.home.html; contig sequences, from The Gene Index Project (http://compbio.dfci.harvard.edu/index.html). All other sequences are from GenBank (http://www.ncbi.nlm.nih.gov/). The multiple sequence alignment was performed with Multialin (http://bioinfo.genopole-toulouse.prd.fr/multalin/multalin.html) and ESPript 2.2 (http://espript.ibcp.fr/ESPript/). (B) Comparisons of Arabidopsis and poplar SHR-like predicted protein sequences. Phylogenetic analysis of closely related predicted amino acid sequences of Arabidopsis and poplar members of the GRAS family [6]. The phylogenetic tree was generated using ClustalW and PHYLIP. The parsimonious tree is shown with bootstrap support values at the nodes. Circles indicate putative poplar-Arabidopsis orthology groups. The Gene model IDs from JGI (Joint Genome Institute) or AGI (Arabidopsis Genome Initiative) ID of each sequence are shown in parentheses. Populus tremula: PtSHR1 (eugene3.01860017), PtSHR2A (eugene3.00070144), PtSHR2B (eugene3.00640143), PtSCL35b (eugene3.00050544), PtSCL53b (eugene3.00640007), PtSCL62 (fgenesh4_pm.C_LG_III000210), PtSCL69b (eugene3.00070272), PtSCL92b (eugene3.00030248), PtSCL97b (eugene3.00011016); Arabidopsis thaliana: AtSHR (At4g37650), AtSCL29 (At3g13840), AtSCL32 (At3g49950). (DOC) [file pone.0028878.s001.doc]

**Supporting Information S1**
