# Supplementary material for: Reduced Expression of the SHORT-ROOT Gene Increases the Rates of Growth and Development in Hybrid Poplar and Arabidopsis
Source: PLoS One. 2011 Dec 14;6(12):e28878. doi: 10.1371/journal.pone.0028878 (PMC3237562; doi:10.1371/journal.pone.0028878)
Supplement: Figure S2 — Relative transcript levels of WT and independent SHR RNAi suppression lines in poplar and Arabidopsis . (A) PtSHR1 RNAi and WT T89 lines. (B) AtSHR RNAi and WT Col0 lines. Real-time RT-PCR was used to compare steady-state transcript levels in WT and PtSHR1 and AtSHR in poplar and Arabidopsis, respectively. For poplar, the expression level of 26S proteasome regulatory subunit S2 was used as an internal reference to which PtSHR1 expression was normalized. 18S rRNA was used for Arabidopsis. (DOC) [file pone.0028878.s002.doc]

**Supporting Information S2**
