# Supplementary material for: Reduced Expression of the SHORT-ROOT Gene Increases the Rates of Growth and Development in Hybrid Poplar and Arabidopsis
Source: PLoS One. 2011 Dec 14;6(12):e28878. doi: 10.1371/journal.pone.0028878 (PMC3237562; doi:10.1371/journal.pone.0028878)
Supplement: Figure S3 — Comparison of fibre widths (A) and lengths (B) comparing independent PtSHR1 RNAi (Lines 2A, 2B) and WT T89 lines. Widths and lengths of fibres extracted from fully elongated internodes of actively growing 2 month old glasshouse grown poplar stems. Means±S.E.M. (n = 60). All P>0.05 ANOVAs followed by Dunnett's posthoc test. (DOC) [file pone.0028878.s003.doc]

**Supporting Information S3**
