# Supplementary material for: Reduced Expression of the SHORT-ROOT Gene Increases the Rates of Growth and Development in Hybrid Poplar and Arabidopsis
Source: PLoS One. 2011 Dec 14;6(12):e28878. doi: 10.1371/journal.pone.0028878 (PMC3237562; doi:10.1371/journal.pone.0028878)
Supplement: Figure S4 — Comparison of fully expanded leaf area (A) and fresh weight (B) comparing independent PtSHR1 RNAi (Lines 2A, 2B, 4A) and WT T89 lines at 52 days growth in the glasshouse. Means ±S.E.M. (n = 9) are the average area and weight of 15 fully expanded leaves from 20 cm above the soil. All P>0.05 ANOVAs followed by Dunnett's posthoc test. (DOC) [file pone.0028878.s004.doc]

**Supporting Information S4**
