# Supplementary material for: Reduced Expression of the SHORT-ROOT Gene Increases the Rates of Growth and Development in Hybrid Poplar and Arabidopsis
Source: PLoS One. 2011 Dec 14;6(12):e28878. doi: 10.1371/journal.pone.0028878 (PMC3237562; doi:10.1371/journal.pone.0028878)
Supplement: Figure S5 — Comparison of shr mutant with Col0 WT leaf characteristics. (A) Average leaf epidermal cell area. (B) Total leaf area of fully expanded leaf 4. Means ±S.E.M. Cell area P>0.05 (n = 20); Leaf area P<0.01 (n = 10). (DOC) [file pone.0028878.s005.doc]

**Supporting Information S5**
