# Supplementary material for: Reduced Expression of the SHORT-ROOT Gene Increases the Rates of Growth and Development in Hybrid Poplar and Arabidopsis
Source: PLoS One. 2011 Dec 14;6(12):e28878. doi: 10.1371/journal.pone.0028878 (PMC3237562; doi:10.1371/journal.pone.0028878)
Supplement: Figure S6 — promPtSHR1 (2.5 kb)-driven eGFP expression in poplar (T89) roots. (A) Expression in stele at root tip. (B) (C) In developing lateral roots. Propidium iodide (red) used as a counterstain. (DOC) [file pone.0028878.s006.doc]

**Supporting Information S6**
