# Supplementary material for: Reduced Expression of the SHORT-ROOT Gene Increases the Rates of Growth and Development in Hybrid Poplar and Arabidopsis
Source: PLoS One. 2011 Dec 14;6(12):e28878. doi: 10.1371/journal.pone.0028878 (PMC3237562; doi:10.1371/journal.pone.0028878)
Supplement: Figure S7 — promAtSHR1 (2.5 kb)-driven GUS expression in Arabidopsis plants. (A) Primary root with developing lateral root (circled). (B) Cotyledons and hypocotyl of seedling 16 hours after germination. (C) Fully expanded juvenile rosette leaf. (D) Flower. (DOC) [file pone.0028878.s007.doc]

**Supporting Information S7**
