# Supplementary material for: Reduced Expression of the SHORT-ROOT Gene Increases the Rates of Growth and Development in Hybrid Poplar and Arabidopsis
Source: PLoS One. 2011 Dec 14;6(12):e28878. doi: 10.1371/journal.pone.0028878 (PMC3237562; doi:10.1371/journal.pone.0028878)
Supplement: Figure S8 — Growth of F1 (WT×WT) and (WT× shr ) seedlings. (A) After 40 hours growth. (B) After 6 DAS. (C) After 15 DAS. (DOC) [file pone.0028878.s008.doc]

**Supporting Information S8**
