# Supplementary material for: Reduced Expression of the SHORT-ROOT Gene Increases the Rates of Growth and Development in Hybrid Poplar and Arabidopsis
Source: PLoS One. 2011 Dec 14;6(12):e28878. doi: 10.1371/journal.pone.0028878 (PMC3237562; doi:10.1371/journal.pone.0028878)
Supplement: Table S1 — t-tests showing mean width differences between VC of RNAi lines 2A, 2B, 4A and WT T89. Measurements were taken from images of plastic embedded transverse sections of young stems (18 internodes down from youngest visible leaf primordium) (* P<0.05; ** P<0.01). (DOC) [file pone.0028878.s009.doc]

**Table S1.** *t*-tests showing mean width differences between VC of RNAi lines 2A, 2B, 4A and WT T89. Measurements were taken from images of plastic embedded transverse sections of young stems (18 internodes down from youngest visible leaf primordium) (^*^*P* < 0.05; ^**^*P* < 0.01).

| Line | *M* | *SD* | *t-value* | *n* |
| --- | --- | --- | --- | --- |
| 2A | 81 | 36.22 | 1.46 | 6 |
| 2B | 104.57^*^ | 42.93 | 2.65 | 7 |
| 4A | 89.17^*^ | 16.51 | 3.79 | 6 |
| T89 | 83.62 | 32.78 | / | 7 |
